# The utility of pericapsular nerve group (PENG) block versus intrathecal morphine for postoperative analgesia in anterior approach total hip arthroplasty: a multicentre triple blinded randomised controlled trial protocol

**DOI:** 10.1186/s13063-025-09127-8

**Published:** 2025-09-29

**Authors:** Craig Morrison, Tim Cheok, Brigid Brown, Bronwyn Lock, Gemma Saville, Louise de Prinse, Holly Alford, Marni Calvert, Dimitrios Nikos, Nikki May, Hidde M. Kroon, Ruurd Jaarsma, Ki Jinn Chin, D.-Yin Lin

**Affiliations:** 1 College of Medicine and Public Health, Flinders University, Bedford Park, Adelaide, SA Australia; 2 Department of Anesthesiology, Flinders Medical Centre, Bedford Park, Adelaide, SA Australia; 3 Department of Orthopaedic Surgery, Flinders Medical Centre, Bedford Park, Adelaide, SA Australia; 4 Gus Fraenkel Medical Library, Flinders University, Bedford Park, Adelaide, SA Australia; 5 Department of Surgery, Royal Adelaide Hospital, Adelaide, South Australia Australia; 6 Discipline of Surgery, Faculty of Health and Medical Science, School of Medicine, University of Adelaide, Adelaide, SA Australia; 7 Department of Anesthesiology and Pain Medicine, Toronto Western Hospital, University of Toronto, Toronto, Canada

**Keywords:** Total hip replacement, Total hip arthroplasty, PENG block, Pericapsular nerve group block, Regional anaesthesia, Intrathecal morphine, Analgesia

## Abstract

**Background:**

The pericapsular nerve group (PENG) block is a novel regional technique for hip analgesia. Traditionally, intrathecal morphine has been administered for analgesia in hip fracture surgery. Compared with intrathecal morphine, the PENG block may provide superior or equivalent pain score reduction with a more favourable side effect profile and superior patient satisfaction.

**Methods:**

This is a multicentre blinded randomised controlled trial (RCT) that is being conducted at present at two large teaching institutions in Australia. The pericapsular nerve group block (PENG block) shall be compared to low-dose intrathecal morphine for analgesic effect in elective total Hip arthroplasty via the direct anterior approach. Primary outcome shall be dynamic pain score evaluation at 3 and 24 h postoperatively.

**Discussion:**

This is the protocol of our RCT which is currently in the early stages of active recruitment.

**Trial registration:**

Ethics approval was obtained from the Southern Area Local Health Network prior to recruitment of the first patient. This trial was prospectively registered prior to initiation on the Australian and New Zealand Clinical Trials Registry. Australian New Zealand Clinical Trial Registry, ACTRN12623001309673P, 15/12/23, https://www.anzctr.org.au/Trial/Registration/TrialReview.aspx?id=386688&showOriginal=true&isReview=true.

**Supplementary Information:**

The online version contains supplementary material available at 10.1186/s13063-025-09127-8.

## Introduction

Total hip arthroplasty (THA) is one of the most successful operations of the century [1]. In Australia, it has been estimated that the rate of Hip arthroplasty is approximately 171 per 100,000 population [2]. Based on data collected by the Australian Orthopaedic National Joint Replacement Registry, the anterior approach to THA constituted approximately 28% of all THAs performed between 2015 and 2022 [3]. The anterior approach is increasingly popular amongst arthroplasty surgeons [4], as it has been associated with lower rates of revision for infection and instability [3].

THA is associated with significant postoperative pain and opioid consumption [5]. Hence, optimisation of perioperative pain management in these patients via a multimodal approach is vital to facilitate early mobilisation and discharge [6], whilst reducing the incidence of postoperative complications [7–9]. Current procedure-specific postoperative pain management (PROSPECT) guidelines for THA advocate for anaesthesiologists to consider 100 µg of intrathecal morphine as a component of spinal anaesthesia for the provision of perioperative analgesia [10]. Although effective as an analgesic, intrathecal morphine has been associated with adverse reactions, such as nausea, vomiting and pruritus, which in turn, may delay mobilisation [11].

The pericapsular nerve group (PENG) block was first described by Girón-Arango et al. in 2018 for regional hip anaesthesia [12]. It is usually thought to be purely sensory blockade, although this is not always the case [9]. Previous studies have shown that both PENG block and intrathecal morphine provided superior analgesic outcomes when compared to placebo; however, there have been no comparative studies between these two modalities [8, 13]. In our study, we will compare the PENG block to intrathecal morphine, which is the current standard of care in our institution, for the provision of perioperative analgesia, measured using numerical pain scale at 3 and 24 h postoperatively, in patients undergoing anterior approach THA.

## Methods

This is a multicentre triple-blinded pragmatic parallel design randomised controlled superiority trial that will be conducted at two teaching hospitals, Noarlunga Health Services (NHS) and Flinders Medical Centre (FMC), both located in Adelaide, Australia. We have obtained ethical approval from the relevant Human Research Ethics Committee prior to begin and shall obtain written informed consent from all participants. This study will conform to the recommendations of Consolidated Standards of Reporting Trials (CONSORT). All researchers have a current Good Clinical Practice certificate from within the last 3 years.

### Patient population

All adult patients above the age of 18 years old presenting at these two large tertiary academic hospitals for primary elective anterior approach THA under spinal anaesthesia will be invited to participate in this study. Included participants must be able to provide informed consent and reliably report symptoms to the research team. We will exclude patients who are (1) unable to provide first party consent (e.g. due to cognitive impairment or language barrier), (2) procedure not performed via an anterior approach, (3) contraindications for any of the following: spinal, intrathecal morphine or PENG block (e.g. therapeutic anticoagulation, anaphylaxis to local anaesthetics or morphine), (4) patient refusal of spinal anaesthesia and/or regional analgesia, (5) clinical indications for general anaesthesia rather than spinal anaesthesia and (6) THA performed for pathological lesions.

All patients scheduled for primary elective direct anterior approach Hip arthroplasty will be approached for potential inclusion during their preoperative assessment at preadmission clinic or immediately afterwards. This strategy is to maximise recruitment opportunities; we estimated that we can achieve our targeted trial enrolment in 8 months based on historical data and have included a contingency for an additional 1-month recruitment in our trial. This is normally held 2–4 weeks preoperatively. Patients can consent to participate up until the day of surgery itself. Consent will be undertaken by a trained research nurse separate to the patients surgical and anaesthetic providers. It will be stressed that non-participation will not alter their treatment and consent can be withdrawn at any point in time. A patient information sheet will also be provided. Participants will be given written information, the opportunity to ask questions and the right to withdraw at any time without consequence. Participant retention in the trial is expected to be high due to the short-term nature of in-hospital only follow-up. Descriptive data will be collected on any reason for participant discontinuation and protocol deviations.

### Study intervention

Anaesthetic technique will be standardised to a spinal anaesthesia with 0.5% isobaric bupivacaine (range 10–15 mg), followed by a single intravenous dose of 8 mg dexamethasone. Following the administration of spinal anaesthesia, the allocated analgesic technique was performed. In patients allocated to the intrathecal morphine group, a sham PENG block will be performed. However, in the PENG group, a sham injection of intrathecal morphine will not be added. Prior to patient inclusion in the trial, it will be confirmed that no contraindications to either technique exist such that there will be no need to modify allocated interventions for individual participants. A research nurse with a copy of the trial protocol will be present on the day of surgery for each participant to ensure compliance with the study protocol and a senior investigator allocated to be available for all trial queries. The research nurse will maintain blinding by leaving theatre during the administration of the trial intervention.

#### PENG group

The block will be placed using ultrasound guidance with a curvilinear probe (2.5–5 MHz). Twenty millilitres of ropivacaine 0.5% (100 mg) prepared by the anaesthesiologist performing the block will be used. The area is aseptically prepped and draped. The curvilinear probe will be placed transversely, medial to the anterior inferior iliac spine with the medial end of the probe rotated in a caudad direction to align to the superior pubic ramus. A 100-mm Sonoplex needle will be inserted in-plane under ultrasound guidance. Twenty millilitres of local anaesthetic will be injected as a plane block between the psoas fascia and superior pubic rami. A cross shall then be drawn on the skin with surgical marker to disguise the presence of a puncture site.

#### Intrathecal morphine group

In addition to the spinal anaesthesia already infiltrated, a further 100 mcg of morphine will be administered. The sham block will be then simulated by the anaesthesiologist by prepping, scanning and draping as per PENG block protocol. The probe and a blunt needle, with a 20-mL syringe filled with saline attached, were held against the skin, similar to the PENG block, and a sufficient pause to simulate the block being performed will be conducted, without penetration of skin or actual administration of any medicine. Likewise, a cross shall then be drawn on the skin with surgical marker to disguise the absence of a puncture site.

### Preoperative education, surgical technique and postoperative management

Preoperatively, patients will be educated on the procedure and expected postoperative rehabilitation. On the day of surgery, following the allocated anaesthesia regimen, the patient is positioned supine, prepped and draped for surgery. Prophylactic antibiotics and tranexamic acid (TXA) will be administered prior to skin incision. The surgery will be conducted by a fellowship-trained orthopaedic surgeon utilising the direct anterior approach. Preparation of the acetabulum and femur will be performed, followed by trial and definitive implant insertion. Routine pericapsular local infiltration analgesia will be performed in all patients, utilising 100 mL of 0.2% ropivacaine with 1 mg adrenaline. Postoperative analgesia regime was standardised with regular paracetamol and NSAIDs if no contraindication and if needed tramadol, oxycodone and/or fentanyl on a nurse-administered basis. Additionally, postoperative antiemetics and antihistamines are also administered as required. All patients will receive two further doses of postoperative antibiotics and allowed to mobilise as tolerated. The urinary catheter will be removed within 24 h unless the patient has a long-term urinary catheter.

### Data collection and outcomes of interest

**Fig. 1:**
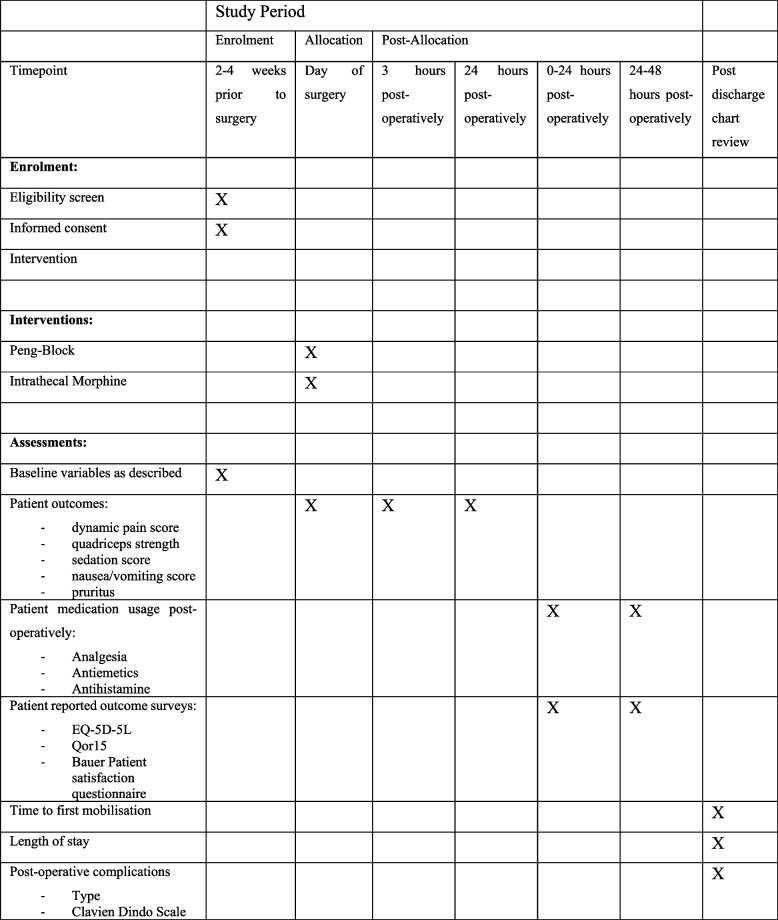
SPIRIT formatted display of schedule for enrolment, intervention and assessments timing

Baseline demographic information including age, gender, ethnicity, body mass index, type of residence, chronic opioid usage, residence type, ASA grade, Charlson Comorbidity Index, preoperative patient reported outcome measures (Oxford Hip Score, EQ-5D-5L Index, pain score), preoperative mobility status and comorbidities will be collected prior to randomisation. The type of prosthesis used, as well as intraoperative complications will be recorded accordingly. Overall evaluation is expected to take approximately 30 min of the participants’ time. No compensation will be offered. Timing of data collection is shown in Fig. 1. Data entry into the password-protected trial database will be performed by trained research nurses and checked by senior investigators. In accordance with institutional policy, data will be stored for 15 years on the secure hospital server with access available to senior investigators.

#### Primary outcomes

The primary outcome of interest is the dynamic pain score at 3 and 24 h postoperatively. These scores are measured using a numerical pain scale from zero to ten.

#### Secondary outcomes

See Fig. 2.

**Fig. 2:**
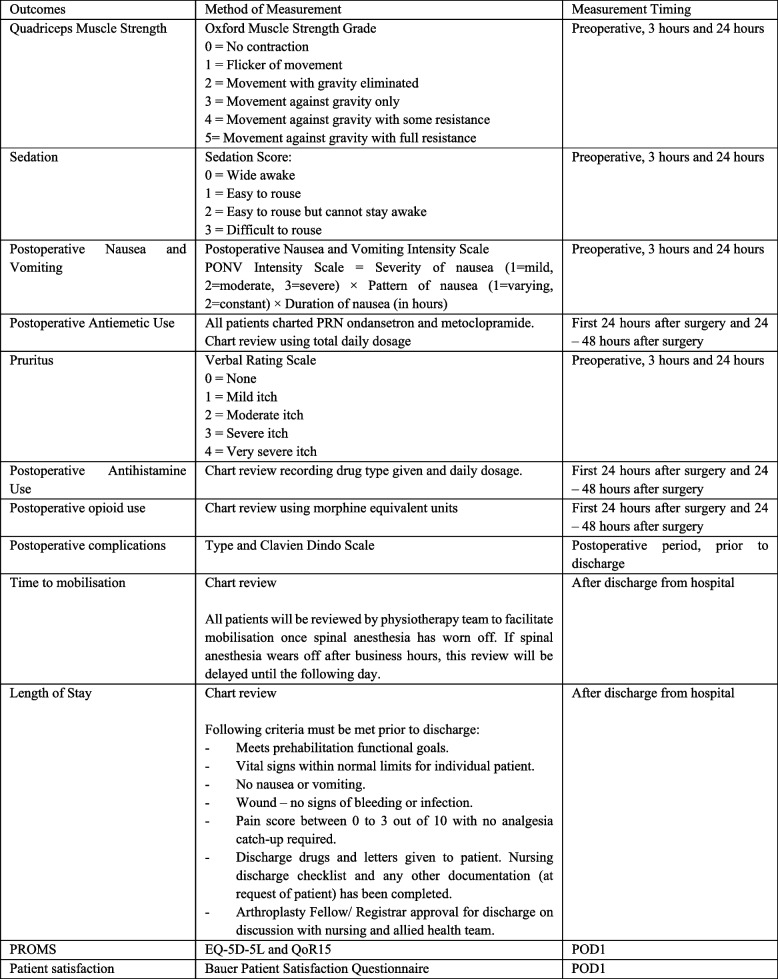
Secondary outcomes

### Recruitment, enrolment and randomisation

Patient recruitment will be performed by a research nurse, who would identify potential participants, as well as screening them against the eligibility criteria. Informed consent would then be obtained by the research team following discussions about the expected benefits, risks, trial commitments and uncertainties of each technique. This is separate to the surgical consent for the procedure itself.

Computer-generated random treatment assignment (sealedenvelope.com) on a 1:1 ratio with permutated blocks (randomly generated blocks of 2, 4 and 6) will be performed immediately prior to surgery by the anaesthetist. Allocation concealment will thus be maintained. The intervention will be blinded to the patient, surgical and treating team, and the research team involved in data collection. Ward nursing staff shall also be blinded and shall be advised to treat all study patients as if they have had intrathecal morphine for safety reasons. Only the anaesthetist performing the intervention and their assistant shall not be blinded.

This is a pragmatic study designed for high external generalisability. All relevant concomitant care deemed necessary by the treating physicians will be permitted and recorded. Participant retention is by nature expected to be high in this RCT as it is short-term perioperative only.

### Sample size calculation

We performed a retrospective case note review to determine the effect size required. As we had two primary outcomes, the larger of the two sample size calculations was utilised. Sample size calculation was then performed using G*Power (Düsseldorf, Germany) with two-sided significance level of 0.05 and 80% power. The sample size required to detect a mean difference in pain scores of 0.8 at 3 h postoperatively was 26 in each group, whereas that required to detect a mean difference in pain scores of 3.3 at 24 h postoperatively was 20 in each group. Accounting for 30% dropout or non-adherence rate, we arrived at a final sample size of 35 in each arm.

### Statistical analysis

All statistical analyses will be performed using Stata version 18 (StataCorp, USA). The threshold for statistical significance in this study is set at 5%. As two primary outcomes are measured, a corrected *p* value threshold of 0.025, obtained using the Bonferroni correction technique, will be utilised when assessing these outcomes. Intention to treat analysis will be performed in all cases. Categorical variables will be displayed as frequency (percentage) and analysed using chi-squared test. Continuous variables will be assessed for normality using the Shapiro–Wilk test. Normally distributed data will be displayed as mean ± standard deviation and analysed using unpaired *t*-test. Continuous data that is not normally distributed will be displayed as median (range) and analysed using Mann–Whitney *U* test. Nonadherence to randomly assigned treatment may mean that the intention to treat analysis may misrepresent the true treatment effect. Therefore, a per-protocol analysis will be performed if there is greater than 10% crossover between groups. We do not plan on undertaking an interim data analysis due to the small sample size of the trial and the use of two routine clinical interventions.

## Benefit and risks in participation

There is no guarantee of any individual benefit in taking part in this study. There is potential that the PENG block may work better in providing pain relief and have a lesser side effect profile, although it has yet to be definitively proven.

Both techniques are widely used in clinical practice, and as such significant harms are unlikely. The side effects of local anaesthesia may include allergic reaction, bleeding, infection, permanent nerve damage and systemic toxicity, whereas the side effects of morphine include allergy, respiratory depression, pruritus, nausea and vomiting.

Where significant harm has occurred, the senior investigators (Prof Jaarsma, Dr Lin, Dr Cheok) will perform an unblinding process to identify which treatment drug was provided. This will be considered on a case-by-case basis. We anticipate that the risk of this occurring would be minimal. Criteria for discontinuing or modifying allocated interventions for a given trial participant include but are not limited to drug dose change in response to harms, participant request or uncontrollable perioperative pain.

## Privacy and data storage

All identifiable or re-identifiable information will remain confidential and will be kept under secure conditions in the Orthopaedic Department at Flinders Medical Centre. All patient’s names will be converted into a number, with only a key available to the senior investigators (Prof Jaarsma, Dr Lin, Dr Cheok). Individual data will not be presented. All data will be destroyed after 15 years.

## Dissemination of results

It is anticipated that the results of this research project will be published and/or presented in a variety of forums, including peer-reviewed medical journals and medical conferences. In any publication and/or presentation, information will be provided in such a way that the data is not re-identifiable. In accordance with relevant Australian and South Australian privacy and other relevant laws, participants have the right to request access to their information collected and stored by the research team. Participants also have the right to request that any disagreeable information is corrected. A copy of any published research, as well as a lay-person summary, will be provided at the request of participants.

## Trial status

Trial protocol version 3.0 last update 30/03/2025. Recruitment commenced February 2025, expected completion August 2025.

## Timeline

Conceptualisation 2023. Funding application, trial registration, ethics application 2024. Trial recruitment 2025. Expected dissemination of results late 2025, early 2026.

## Supplementary Information

Additional file 1: SPIRIT checklist.
